# Leptin signalling altered in infantile nephropathic cystinosis‐related bone disorder

**DOI:** 10.1002/jcsm.13579

**Published:** 2024-08-29

**Authors:** Wai W. Cheung, Ping Zhou, Ronghao Zheng, Arieh Gertler, Eduardo A. Oliveira, Robert H. Mak

**Affiliations:** ^1^ Division of Pediatric Nephrology, Rady Children's Hospital University of California, San Diego La Jolla CA USA; ^2^ Department of Pediatric Nephrology and Rheumatology, Sichuan Provincial Maternity and Child Health Care Hospital Sichuan Clinical Research Center for Pediatric Nephrology and The Affiliated Women's and Children's Hospital of Chengdu Medical College Chengdu China; ^3^ Department of Pediatric Nephrology, Rheumatology, and Immunology, Maternal and Child Health Hospital of Hubei Province, Tongji Medical College Huazhong University of Science and Technology Wuhan China; ^4^ School of Biological and Population Health Sciences, Institute of Biochemistry, Food Science and Nutrition Hebrew University of Jerusalem Rehovot Israel; ^5^ Department of Pediatrics, Division of Pediatric Nephrology, Faculty of Medicine Federal University of Minas Gerais (UFMG) Belo Horizonte Brazil

**Keywords:** AgRP, Bone, Infantile nephropathic cystinosis, Leptin, Melanocortin receptor

## Abstract

**Background:**

The *CTNS* gene mutation causes infantile nephropathic cystinosis (INC). Patients with INC develop Fanconi syndrome and chronic kidney disease (CKD) with significant bone deformations. C57BL/6 *Ctns* ^*−/−*^ mice are an animal model for studying INC. Hyperleptinaemia results from the kidney's inability to eliminate the hormone leptin in CKD. *Ctns* ^*−/−*^ mice have elevated serum leptin concentrations. Leptin regulates bone metabolism through its receptor that signals further via the hypothalamic melanocortin 4 receptor (MC4R). Leptin signalling may affect bone health in *Ctns* ^*−/−*^ mice.

**Methods:**

We first defined the time course of bone abnormalities in *Ctns* ^*−/−*^ mice between 1 and 12 months of age. We used both genetic and pharmacological approaches to investigate leptin signalling in *Ctns* ^*−/−*^ mice. We generated *Ctns* ^*−/−*^ *Mc4r* ^*−/−*^ double knockout mice. Bone phenotype of *Ctns* ^*−/−*^ *Mc4r* ^*−/−*^ mice, *Ctns* ^*−/−*^ mice and wild type (WT) mice at 1, 4, and 9 months of age were compared. We then treated 12‐month‐old *Ctns* ^*−/−*^ mice and WT mice with a pegylated leptin receptor antagonist (PLA) (7 mg/kg/day, IP), a MC4R antagonist agouti‐related peptide (AgRP) (2 nmol, intracranial infusion on days 0, 3, 6, 9, 12, 15, 18, 21, 24, and 27), or vehicle (normal saline), respectively, for 28 days. Whole‐body (BMC/BMD, bone area) and femoral bone phenotype (BMC/BMD, bone area, length and failure load) of mice were measured by DXA and femoral shaft biochemical test. We also measured lean mass content by EchoMRI and muscle function (grip strength and rotarod activity) in mice. Femur protein content of JAK2 and STAT3 was measured by ELISA kits, respectively.

**Results:**

Bone defects are present in *Ctns* ^*−/−*^ mice throughout its first year of life. The deletion of the *Mc4r* gene attenuated bone disorder in *Ctns* ^*−/−*^ mice. Femoral BMD, bone area, length, and strength (failure load) were significantly increased in 9‐month‐old *Ctns* ^*−/−*^ *Mc4r* ^*−/−*^ mice than in age‐matched *Ctns* ^*−/−*^ mice. PLA and AgRP treatment significantly increased femoral bone density (BMC/BMD) and mechanical strength in 12‐month‐old *Ctns* ^*−/−*^ mice. We adopted the pair‐feeding approach for this study to show that the protective effects of PLA or AgRP on bone phenotype are independent of their potent orexigenic effect. Furthermore, an increase in lean mass and in vivo muscle function (grip strength and rotarod activity) are associated with improvements in bone phenotype (femoral BMC/BMD and mechanical strength) in *Ctns* ^*−/−*^ mice, suggesting a muscle‐bone interplay. Decreased femur protein content of JAK2 and STAT3 was evident in *Ctns* ^*−/−*^ mice. PLA or AgRP treatment attenuated femur STAT3 content in *Ctns* ^*−/−*^ mice.

**Conclusions:**

Our findings suggest a significant role for dysregulated leptin signalling in INC‐related bone disorder, either directly or potentially involving a muscle‐bone interplay. Leptin signalling blockade may represent a novel approach to treating bone disease as well as muscle wasting in INC.

## INTRODUCTION

Infantile nephropathic cystinosis (INC) is a metabolic disorder caused by cystine accumulating in lysosomes.^1^ *CTNS*, the gene encoding cystinosin, or the cystine transporter, is mutated. Patients with INC exhibit Fanconi syndrome and chronic kidney disease (CKD).^2^ In addition, INC patients also display significant bone deformities,^2^, ^3^ which are significantly worse than patients with other causes of CKD.^4^ The pathophysiology of bone complications in INC is poorly understood, as they occur before the onset of tubular and glomerular dysfunction. Cysteamine therapy is the cornerstone of management for INC patients as it reduces lysosomal cystine accumulation in target organs^2^ but is not associated with improvement in bone symptoms.^5^

The neuroendocrine hormone leptin regulates bone metabolism and neuroendocrine function.^6^ Leptin's actions are mediated through the leptin receptors that are found throughout various peripheral organs and tissues including bone.^6^ Both animal and human studies have shown that leptin affects bone metabolism via direct and indirect pathways.^7^ Leptin is degraded in the kidneys and hyperleptinaemia is common in CKD patients.^8^ Our recent published data show that serum leptin concentration was significantly elevated in C57BL/6 *Ctns* ^*−/−*^ mice, an animal model for studying INC.^9^ Leptin activates its receptor that signals through the melanocortin 4 receptor (MC4R) in the hypothalamus to influence appetite, metabolic rate, and bone metabolism.^6^ *Mc4r* ^*−/−*^ mice display increased bone mass and strength. A loss‐of‐function mutation in the *MC4R* results in an increase in total body bone mineral content (BMC) and bone mineral density (BMD) in patients,^10^ suggesting that leptin regulates bone mass through hypothalamic MC4R signalling. In this study, we examine whether leptin signalling blockade improves bone abnormalities in *Ctns* ^*−/−*^ mice.

## Methods

### Experimental design

To test our hypothesis, we utilized congenic C57BL/6 *Ctns* ^*−/−*^ mice, initially provided by Professor Corinne Antignac. Wild‐type (WT) C57BL/6 breeder mice were purchased from The Jackson Lab. C57BL/6 *Ctns* ^*−/−*^ mice and WT mice were from the same C57BL/6 genetic background. We generated in house male C57BL/6 *Ctns* ^*−/−*^ mice and male WT C57BL/6 mice for this study. The mice were housed in 12:12 h light–dark cycles with ad libitum access to mouse diet 5,015 (LabDiet, catalogue 0001328). We followed established guidelines and standards when conducting this study. In accordance with the guidelines of the National Institutes of Health, IACUC approved protocol S01754 at the University of California, San Diego.

Four experiments were conducted. Study 1: A study was conducted to define the time course of bone abnormalities in *Ctns* ^*−/−*^ mice between 1 and 12 months of age with respect to kidney function abnormalities. The key parameters of bone phenotype (whole body BMC/BMD and bone area) in *Ctns* ^*−/−*^ mice were compared with WT mice at 1, 4, 9, and 12 months of age. To further investigate skeletal integrity in *Ctns* ^*−/−*^ mice, excised left femurs were used for appendicular BMC/BMD/bone area, length, and mechanical strength (failure load) measurements. Study 2: We defined the significance of melanocortin signalling in cystinosis bone disease by a genetic approach. We generated *Ctns* ^*−/−*^ *Mc4r* ^*−/−*^ mice by crossing *Ctns* ^*−/−*^ mice with *Mc4r* ^*−/−*^ mice. Roger Cone's laboratory provided C57BL/6 *Mc4r* ^*−/−*^ mice.^11^ *Ctns* ^*−/−*^ *Mc4r* ^*−/−*^ mice, *Ctns* ^*−/−*^and WT mice were on the same C57BL/6 genetic background. Parameters of whole‐body and femoral bone phenotype in *Ctns* ^*−/−*^ *Mc4r* ^*−/−*^ mice were compared with *Ctns* ^*−/−*^ mice and WT mice at 1, 4, and 9 months of age. Study 3: We tested the significance of leptin signalling in cystinosis bone disease pharmacologically. Pegylated leptin receptor antagonists (PLA) bind to leptin receptors without activating them. Professor Arieh Gertler prepared and provided the PLA.^12^ Twelve‐month‐old male *Ctns* ^*−/−*^ mice and WT mice were given PLA (7 mg/kg/day, IP) or vehicle (normal saline) for 28 days. We used a pair‐feeding strategy. Vehicle injected *Ctns* ^*−/−*^ mice were fed ad libitum. Dietary intake of vehicle‐treated *Ctns* ^*−/−*^ mice was recorded. PLA‐treated *Ctns* ^*−/−*^ mice and WT mice injected with PLA or vehicle received an average daily intake of the vehicle‐treated *Ctns* ^*−/−*^ mouse diet. Whole body bone parameters and femoral bone parameters, as listed in Study 1, were measured. Study 4: We evaluated the impact of melanocortin signalling on cystinosis bone disease pharmacologically. Agouti‐ related peptide (AgRP) is a hypothalamic neuropeptide which antagonizes leptin signalling.^13^ We tested AgRP response in mice. We implanted cannulations into 12‐month‐old *Ctns* ^*−/−*^ mice and and WT mice. A 10‐μL Hamilton syringe was used to inject 2 nmol of AgRP (82–131 amino acid fragment, Phoenix Pharmaceuticals) or normal saline (vehicle) into the lateral ventricles of mice.^13^ AgRP or vehicle was infused into mice on days 0, 3, 6, 9, 12, 15, 18, 21, 24, and 27. This study was also conducted using a pair‐feeding strategy. The vehicle‐treated *Ctns* ^*−/−*^ mice were fed ad libitum. In contrast, AgRP‐infused *Ctns* ^*−/−*^ mice and AgRP‐treated or vehicle‐treated WT mice were given the same amount of the rodent diet as consumed by the vehicle‐treated *Ctns* ^*−/−*^ mice. We evaluated parameters of bone phenotype in mice.

### In vivo whole‐body composition analysis

An X‐ray densitometer (GE Medical Systems, Chicago, IL, USA) was used to determine the mice's whole‐body composition using dual‐energy X‐ray absorption (DXA).^11^ DXA analysis was performed on all animals after they had fasted for 12 h to minimize the amount of indigested food. In this study, we measured the bone mineral content (BMC), bone mineral density (BMD), and bone area of mice at the whole‐body level.

### Serum and blood chemistry

Mice were sacrificed after fasting for 4 h. A VetScan2 VS2 Comprehensive Diagnostic Profile (Abaxis, catalogue 500‐0038) was used to measure bicarbonate, calcium, potassium, and blood urea nitrogen concentrations in serum. By using tandem mass spectrometry and liquid chromatography, serum creatinine concentrations were determined. Serum intact PTH levels were measured using Immunotropic, catalogue 60‐2305. Concentrations of serum 25(OH)D_3_, 1,25(OH)_2_D_3_ were also analysed with Immunodiagnostic systems EIA AC‐57SF1 and AC‐62F1. Serum FGF23 and leptin concentration were measured using the mouse FGF23 ELISA kit, catalogue ab213863 and Invitrogen, Mouse Leptin ELISA kit, catalogue KMC 2281.

### Ex vivo femoral bone analysis

Femurs were harvested at necropsy. Measurements of BMC, BMD, and bone area of the left excised femora were performed with a pixiMus™ mouse densitometer (GE Medical Systems, Chicago, IL, USA). Length of the extracted femur was measured using a fractional digital calliper (Carrera Precision).

### Femoral shaft biomechanical test

An Instron Corporation high‐resolution materials test apparatus (Model 4,442, Canton, MA, USA) was used to test the 3‐point bending of excised left femora. There are two fixed lower supports with a span length of 7 mm in the loading fixture. A moving actuator is also attached to the upper loading point. Two lower supports supported the caudal surface of the femur. At the specimen's midpoint, which coincided with the span's centre, the upper loading point was in contact with the specimen. Software (Series IX for Windows 95) displaced the actuator at a strain rate of 0.5%/s until failure occurred. System software was used to collect load and displacement data.

### Lean mass content and muscle function

We measured the lean mass content with a quantitative magnetic resonance analysis (EchoMRI‐100TM, Echo Medical System, Houston, TX, USA).^9^ An assessment of the mice's forelimb grip strength (Model 47106, UGO Basile, Gemonio, Italy) and the activity of rotarods (model RRF/SP, Accuscan Instrument, Columbus, OH, USA) was conducted.^9^

#### Femur protein assay

The right femur was processed in a homogenizer tube (USA Scientific, catalogue 1420‐9600, Ocala, FL, USA) containing ceramic beads (Omni International, catalogue 19‐646, Kennesaw, GA, USA). Total protein from the dissected bone sample was extracted using TriZol (Life Technology, Carlsbad, CA, USA). Protein concentration of tissue homogenate was assayed using Pierce BAC Protein Assay Kit (Thermo Scientific, catalogue 23227, Waltham, MA, USA). Protein contents of JAK2 phosphor‐Tyr221, total JAK2, STAT (pY705)+ total STAT3 in the tissue homogenates were measured by Antibodies‐online.com, catalogue ABIN1380619, Abcam, catalogue ab253224 and Abcam, catalogue ab126459, respectively.

#### Statistics

Prism version 10.1.1 was used for statistical analyses. The data are presented as mean ± SEM. Student's two‐tailed *t*‐tests were used to compare means between groups. Two‐way ANOVA was used to analyse differences between the means of more than two groups containing two variables. Tukey's test was used for post‐hoc analysis. Significant results were defined as *P*‐values below 0.05.

## Results

### The timeline of skeletal phenotype in *Ctns* ^*−/−*^ mice

**Figure 1:**
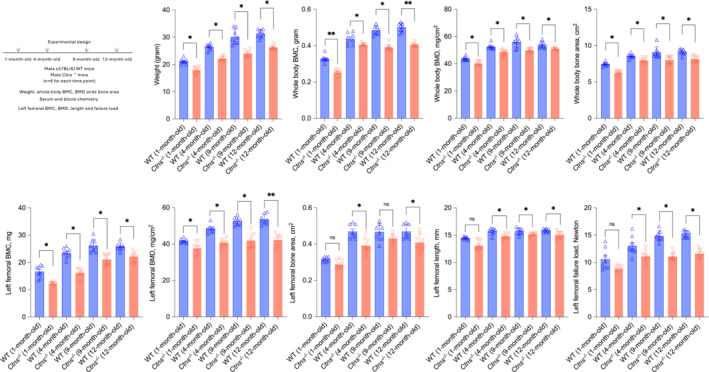
Skeletal phenotype in *Ctns* ^*−/−*^ mice during the 12‐month study. All mice were fed ad libitum. Experimental design is listed. Mice were weighted to the nearest 0.1 g and then anaesthetized for in vivo scanning by dual‐energy X‐ray absorptiometry (DXA) to determine whole bone mineral content (BMC), bone mineral density (BMD) and bone area. The isolated left femora were assessed by DXA. Femoral BMC, BMD and bone area. Femoral length and load to failure were measured. Data are expressed as mean ± SEM. Results of *Ctns* ^*−/−*^ mice were compared with age‐matched WT mice. Ns, not significant, *P < 0.05, **P < 0.01.

We confirmed out previous published data that *Ctns* ^*−/−*^ mice exhibit Fanconi syndrome at 4 months of age and chronic kidney disease at the age of 9 months.^14^, ^15^ To characterize the timeline of bone deformities in *Ctns* ^*−/−*^ mice, we evaluated bone phenotype in *Ctns* ^*−/−*^ mice at 1, 4, 9 and 12 months of age with age‐matched WT mice, respectively. Age‐matched *Ctns* ^*−/−*^ mice and WT mice were sacrificed. Figure 1 shows the experimental design. Table 1 shows the results of serum and blood chemistry. *Ctns* ^*−/−*^ mice displayed hypophosphataemia at 4 months of age. A significant increase in serum contents of BUN and creatinine was observed in 9‐month‐old *Ctns* ^*−/−*^ mice. The increased intact PTH levels in *Ctns* ^*−/−*^ mice relative to WT mice indicate mild hyperparathyroidism in *Ctns* ^*−/−*^ mice. Decreased serum 25(OH)D_3_ and 1,25(OH)_2_D_3_ was found in 9‐month‐old *Ctns* ^*−/−*^ mice. The bicarbonate and calcium contents of serum were not different between *Ctns* ^*−/−*^ mice and WT mice. Serum FGF23 and leptin levels were elevated in 9‐month‐old and 12‐month‐old *Ctns* ^*−/−*^ mice. As early as 1 month of age, we observed that *Ctns* ^*−/−*^ mice had low bone mass, before the onset of kidney dysfunction. The mean whole‐body BMC/BMD and bone area was significantly lower in *Ctns* ^*−/−*^ mice than in WT mice (Figure 1). Consistent with the lower whole body bone mass phenotype in *Ctns* ^*−/−*^ mice, *Ctns* ^*−/−*^ mice exhibited reduced appendicular (femoral) BMC/BMD relative to WT mice. Femoral bone length and strength (failure load) were not different at 1 month of age, but were significantly decreased in 4‐, 9‐, and 12‐month‐old *Ctns* ^*−/−*^ mice relative to age‐matched WT mice.

**Table 1:** Serum and blood chemistry of mice during the 12‐month study

	WT	*Ctns* ^−/−^	WT	*Ctns* ^−/−^	WT	*Ctns* ^−/−^	WT	*Ctns* ^−/−^
	1‐month‐old (*n* = 8)	1‐month‐old (*n* = 8)	4‐month‐old (*n* = 8)	4‐month‐old (*n* = 8)	9‐month‐old (*n* = 8)	9‐month‐old (*n* = 8)	12‐month‐old (*n* = 8)	12‐month‐old (*n* = 8)
BUN (mg/dL)	24.8 ± 2.1	25.8 ± 1.5	26.9 ± 1.3	25.8 ± 0.6	27.0 ± 1.5	59.2 ± 2.1^a^	28.3 ± 1.9	67.8 ± 7.8^a^
Creatinine (mg/dL)	0.06 ± 0.02	0.05 ± 0.02	0.07 ± 0.01	0.07 ± 0.01	0.07 ± 0.01	0.11 ± 0.02^a^	0.09 ± 0.01	0.18 ± 0.02^a^
Bicarbonate (mg/dL)	25.8 ± 2.3	26.8 ± 1.6	27.5 ± 0.5	27.3 ± 1.2	27.2 ± 0.5	27.1 ± 0.4	28.8 ± 1.4	26.9 ± 0.7
Calcium (mg/dL)	9.53 ± 0.12	9.27 ± 0.26	9.62 ± 0.10	10.02 ± 0.09	9.32 ± 0.11	9.21 ± 0.05	9.43 ± 0.12	9.49 ± 0.05
Phosphorus (mg/dL)	9.54 ± 0.21	9.27 ± 0.16	9.60 ± 0.32	8.20 ± 0.26^b^	8.65 ± 0.16	7.76 ± 0.22^b^	8.82 ± 0.31	7.38 ± 0.16^b^
Intact PTH (pg/mL)	90.4 ± 6.3	90.5 ± 4.8	93.8 ± 5.1	101.1 ± 2.7	103.2 ± 10.4	264.6 ± 9.5^a^	118.9 ± 11.7	432.8 ± 12.8^a^
25(OH)D_3_ (ng/mL)	123.2 ± 19.3	101.8 ± 12.9	113.2 ± 15.7	108.2 ± 14.8	120.1 ± 20.5	46.9 ± 8.1^b^	106.5 ± 12.1	50.7 ± 12.6^b^
1,25(OH)_2_D_3_ (pg/mL)	302.4 ± 16.4	252.6 ± 32.7	254.5 ± 17.4	184.4 ± 14.8	276.4 ± 30.6	157.8 ± 19.4^b^	276.8 ± 17.9	115.7 ± 32.6^b^
FGF23 (pg/mL)	102.6 ± 18.3	105.8 ± 21.7	105.2 ± 11.4	113.2 ± 18.4	121.5 ± 20.6	202.1 ± 19.6^a^	108.2 ± 15.2	216.9 ± 22.9^a^
Leptin (ng/dL)	2.21 ± 0.16	2.37 ± 0.12	2.33 ± 0.09	2.34 ± 0.11	2.32 ± 0.05	3.49 ± 0.06^a^	2.49 ± 0.8	5.54 ± 0.12^a^

All mice were fed ad libitum. Data are expressed as mean ± SEM. Results of *Ctns* ^*−/−*^ mice were compared with age‐matched WT mice.

BUN, blood urinary nitrogen; FGF23, fibroblast growth factor 23; PTH, parathyroid hormone.

^a^ *P* < 0.05, significantly higher in *Ctns* ^*−/−*^ mice than WT mice.

^b^ *P* < 0.05, significantly lower in *Ctns* ^*−/−*^ mice than WT mice.

### The deletion of the melanocortin 4 receptor attenuates bone disease in *Ctns* ^−/−^ mice

**Figure 2:**
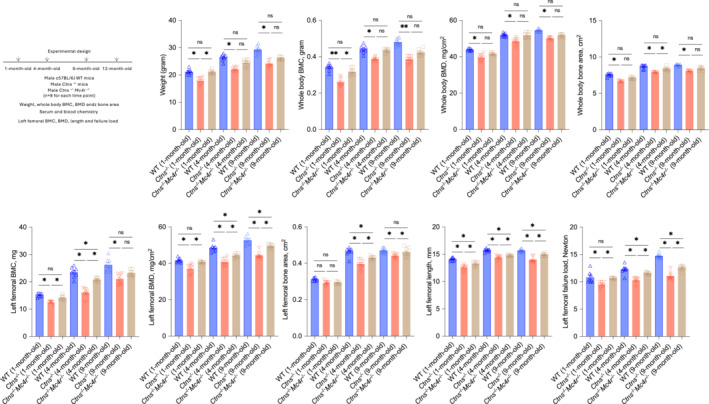
Genetic deletion of *Mc4r* attenuates cystinosis skeletal phenotype in *Ctns* ^*−/−*^ mice. All mice were fed ad libitum. Study design is listed. Body weight and whole‐body bone phenotype as well as femoral bone phenotype was studied as listed in Figure 1. Data are expressed as mean ± SEM. Results of *Ctns* ^*−/−*^ *Mc4r* ^*−/−*^ mice and *Ctns* ^*−/−*^ mice were compared with age‐matched WT mice. Results of *Ctns* ^*−/−*^ *Mc4r* ^*−/−*^ mice were also compared with *Ctns* ^*−/−*^ mice. Ns, not significant, *P < 0.05, **P < 0.01.

Leptin signals through MC4R. The effects of MC4R blockade on bone disease pathology in *Ctns* ^*−/−*^ mice were tested genetically. We generated *Ctns* ^*−/−*^ *Mc4r* ^*−/−*^ mice by crossing *Ctns* ^*−/−*^ and *Mc4r* ^*−/−*^ mice. We compared the bone phenotype of *Ctns* ^*−/−*^ *Mc4r* ^*−/−*^ mice, *Ctns* ^*−/−*^ mice, and WT controls at 1, 4, and 9 months of age. *Ctns* ^*−/−*^ *Mc4r* ^*−/−*^ mice, *Ctns* ^*−/−*^ mice, and WT mice are from the same C57BL/6 genetic background. Figure 2 illustrates the experimental design. Results of serum and blood chemistry in *Ctns* ^*−/−*^ *Mc4r* ^*−/−*^ mice are comparable with those in *Ctns* ^*−/−*^ mice at 1, 4, and 9 months of age (Table 2). Genetic deletion of *Mcr4* improves bone parameters in *Ctns* ^*−/−*^ mice. Femoral BMD, bone area, length, and failure load were significantly increased in 9‐month‐old *Ctns* ^*−/−*^ *Mc4r* ^*−/−*^ mice as compared with age‐matched *Ctns* ^*−/−*^ mice (Figure 2).

**Table 2:** Serum and blood chemistry of mice during the 9‐month study

	WT	*Ctns* ^−/−^	*Ctns* ^−/−^ *Mc4r* ^−/−^	WT	*Ctns* ^−/−^	*Ctns* ^−/−^ *Mc4r* ^−/−^	WT	*Ctns* ^−/−^	*Ctns* ^−/−^ *Mc4r* ^−/−^
	1‐month‐old (*n* = 8)	1‐month‐old (*n* = 8)	1‐month‐old (*n* = 8)	4‐month‐old (*n* = 8)	4‐month‐old (*n* = 8)	4‐month‐old (*n* = 8)	9‐month‐old (*n* = 8)	9‐month‐old (*n* = 8)	9‐month‐old (*n* = 8)
BUN (mg/dL)	29.6 ± 3.7	28.7 ± 2.2	31.7 ± 3.2	30.4 ± 3.6	32.7 ± 3.6	32.6 ± 3.5	30.4 ± 3.2	67.8 ± 9.5 ^a^	72.3 ± 8.6 ^a^
Creatinine (mg/dL)	0.08 ± 0.02	0.10 ± 0.02	0.07 ± 0.02	0.07 ± 0.02	0.12 ± 0.02	0.09 ± 0.02	0.08 ± 0.01	0.016 ± 0.02 ^a^	0.15 ± 0.02 ^a^
Bicarbonate (mg/dL)	27.8 ± 0.4	26.8 ± 1.3	27.9 ± 1.6	27.4 ± 0.7	26.8 ± 0.7	27.7 ± 0.5	27.9 ± 0.9	27.3 ± 1.1	27.6 ± 0.8
Calcium (mg/dL)	9.87 ± 0.15	9.58 ± 0.21	9.49 ± 0.27	9.76 ± 0.27	9.48 ± 0.13	9.85 ± 0.27	9.49 ± 0.33	9.54 ± 0.28	9.39 ± 0.75
Phosphorus (mg/dL)	9.42 ± 0.27	9.65 ± 0.37	9.59 ± 0.43	9.59 ± 0.52	9.07 ± 0.54	8.38 ± 0.48	8.87 ± 0.25	7.37 ± 0.21 ^b^	7.39 ± 0.55 ^b^
Intact PTH (pg/mL)	94.2 ± 13.7	116.5 ± 22.6	14.7 ± 12.7	109.6 ± 25.7	143.9 ± 18.3	135.2 ± 26.3	128.4 ± 25.1	274.1 ± 24.6 ^a^	225.2 ± 21.8 ^a^
FGF23 (pg/mL)	87.1 ± 21.6	79.4 ± 14.8	72.4 ± 6.9	97.2 ± 8.4	106.3 ± 12.7	86.9 ± 11.6	95.7 ± 16.4	221.6 ± 17.8 ^a^	275.4 ± 26.3 ^a^

All mice were fed ad libitum. Data are expressed as mean ± SEM. Results of *Ctns* ^*−/−*^ mice and *Ctns* ^*−/−*^ *Mc4r* ^*−/−*^ mice were compared with age‐matched WT mice.

BUN, blood urinary nitrogen; FGF23, fibroblast growth factor 23; PTH, parathyroid hormone.

^a^ *P* < 0.05, significantly higher in *Ctns* ^*−/−*^ mice or *Ctns* ^*−/−*^ *Mc4r* ^*−/−*^ mice than WT mice.

^b^ *P* < 0.05, significantly lower in *Ctns* ^*−/−*^ mice or *Ctns* ^*−/−*^ *Mc4r* ^*−/−*^ mice than WT mice.

### A pegylated leptin receptor antagonist treatment improves skeletal integrity in *Ctns* ^−/−^ mice

**Figure 3:**
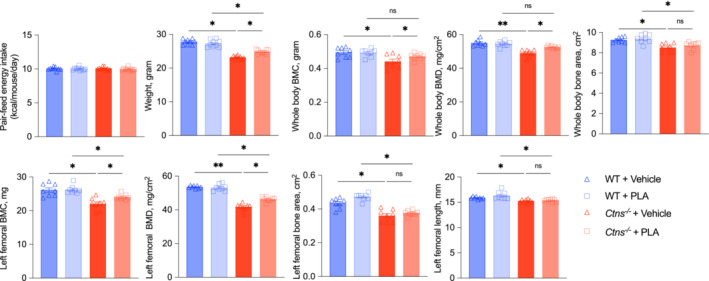
PLA treatment attenuates INC bone disorder in *Ctns* ^*−/−*^ mice. *Ctns* ^*−/−*^ + vehicle mice were fed ad libitum whereas WT + vehicle, WT + PLA and *Ctns* ^*−/−*^ + PLA mice were pair‐fed to that of *Ctns* ^*−/−*^ + vehicle mice. Mice were weighed and scanned for whole‐body BMC, BMD, and bone area. Femoral BMC, BMD, area, and length were also shown. Data are expressed as mean ± SEM. Results of *Ctns* ^*−/−*^ + vehicle mice were compared with that of WT + vehicle mice whereas results of *Ctns* ^*−/−*^ + PLA mice were compared with that of WT + PLA mice. Results of *Ctns* ^*−/−*^ + vehicle mice were also compared with *Ctns* ^*−/−*^ + PLA mice. Ns, not significant, *P < 0.05, **P < 0.01.

We tested whether PLA treatment would improve skeletal integrity in *Ctns* ^*−/−*^ mice. Our published data indicate that PLA stimulates food intake and subsequent weight gain in *Ctns* ^*−/−*^ mice.^9^ To assess the effects of blocking the leptin receptor in *Ctns* ^*−/−*^ mice that go beyond the stimulation of appetite, we adopted a dietary restriction approach. We treated 12‐month‐old *Ctns* ^*−/−*^ mice and WT mice with either PLA (7 mg/kg/day, IP) or vehicle for 28 days. *Ctns* ^*−/−*^ mice injected with vehicles were fed ad libitum and their daily caloric intake were measured. The equivalent amount of rodent diet was then given to *Ctns* ^*−/−*^ mice treated with PLA or WT mice treated with PLA or vehicle (Figure 3). Table 3 shows the results of serum and blood chemistry of mice. Concentration of serum phosphorus was decreased while BUN, creatinine, intact PTH and FGF23 were significantly increased in *Ctns* ^*−/−*^ mice as compared with WT mice. There is no difference in serum bicarbonate and calcium concentrations between *Ctns* ^*−/−*^ mice and WT mice. Leptin serum concentration was significantly higher in *Ctns* ^*−/−*^ mice. The effects of PLA on serum leptin levels were not observed in *Ctns* ^*−/−*^ mice. PLA treatment improved bone disease in *Ctns* ^*−/−*^ mice. Whole‐body weight/BMC/BMD were significantly increased in PLA‐injected *Ctns* ^*−/−*^ mice as compared with *Ctns* ^*−/−*^ mice treated with vehicle (Figure 3). Furthermore, femoral BMC and BMD were considerably increased in *Ctns* ^*−/−*^ mice treated with PLA as compared with *Ctns* ^*−/−*^ mice treated with vehicle. Effects of PLA administration on the femoral failure load in *Ctns* ^*−/−*^ mice are presented in the subsequent Figure 5.

**Table 3:** Serum and blood chemistry of mice

	WT + Vehicle (*n* = 9)	WT + PLA (*n* = 9)	*Ctns* ^*−/−*^ + Vehicle (*n* = 9)	*Ctns* ^*−/−*^ + PLA (*n* = 9)
BUN (mg/dL)	29.5 ± 4.6	32.4 ± 3.1	59.8 ± 6.7^a^	74.5 ± 9.4^a^
Creatinine (mg/dL)	0.09 ± 0.02	0.12 ± 0.04	0.19 ± 0.02^a^	0.21 ± 0.04^a^
Bicarbonate (mg/dL)	28.2 ± 2.2	27.6 ± 2.1	27.3 ± 1.8	27.3 ± 1.1
Calcium (mg/dL)	9.36 ± 0.13	9.54 ± 0.25	9.75 ± 0.21	9.48 ± 0.27
Phosphorus (mg/dL)	8.97 ± 0.21	9.05 ± 0.31	7.76 ± 0.21^b^	7.87 ± 0.21^b^
Intact PTH (pg/mL)	109.6 ± 21.5	143.1 ± 22.6	435.1 ± 37.8^a^	419.5 ± 23.6^a^
FGF23 (pg/mL)	132.1 ± 12.8	105.2 ± 18.2	265.7 ± 34.1^a^	245.3 ± 31.4^a^
Leptin (ng/dL)	2.61 ± 0.31	2.83 ± 0.47	4.57 ± 0.36^a^	5.17 ± 0.29^a^

Twelve‐month‐old *Ctns* ^*−/−*^ mice and WT mice were treated with PLA (7 mg/kg/day, IP) or vehicle (normal saline) for 28 days. Four groups of mice were included: WT + vehicle, WT + PLA, *Ctns* ^*−/−*^ + vehicle and *Ctns* ^*−/−*^ + PLA. *Ctns* ^*−/−*^ + vehicle mice were fed ad libitum whereas WT + vehicle, WT + PLA and *Ctns* ^*−/−*^ + PLA mice were pair‐fed to that of *Ctns* ^*−/−*^ + vehicle mice. Data are expressed as mean ± SEM. Data are expressed as mean ± SEM. Results of *Ctns* ^*−/−*^ + vehicle mice were compared with that of WT + vehicle mice whereas results of *Ctns* ^*−/−*^ + PLA mice were compared with that of WT + PLA mice.

^a^ *P* < 0.05, significantly higher in *Ctns* ^*−/−*^ mice than WT mice.

^b^ *P* < 0.05, significantly lower in *Ctns* ^*−/−*^ mice than WT mice.

### Intracerebroventricular infusion of the agouti‐related peptide improves bone disease in *Ctns* ^−/−^ mice

**Figure 4:**
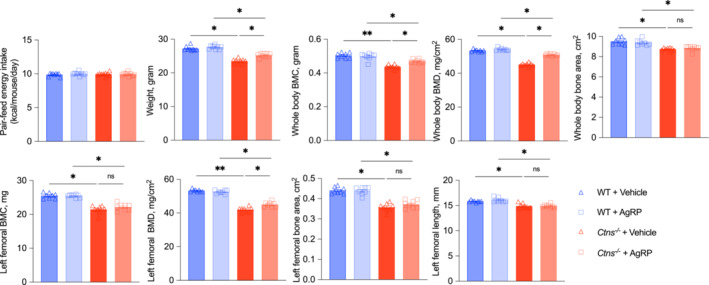
Infusion of AgRP ameliorates INC bone disorder in *Ctns* ^*−/−*^ mice. *Ctns* ^*−/−*^ + vehicle mice were fed ad libitum WT + vehicle, WT + AgRP, and *Ctns* ^*−/−*^ + AgRP mice were pair‐fed to that of *Ctns* ^*−/−*^ + vehicle mice. Whole‐body BMC, BMD, and bone area were measured. Femoral BMC, BMD, area, and length were also shown data are expressed as mean ± SEM. Results of *Ctns* ^*−/−*^ + vehicle mice were compared with that of WT + vehicle mice whereas results of *Ctns* ^*−/−*^ + AgRP mice were compared with that of WT + AgRP mice. Results of *Ctns* ^*−/−*^ + vehicle mice were also compared with *Ctns* ^*−/−*^ + AgRP mice. Ns, not significant, *P < 0.05, **P < 0.01.

**Figure 5:**
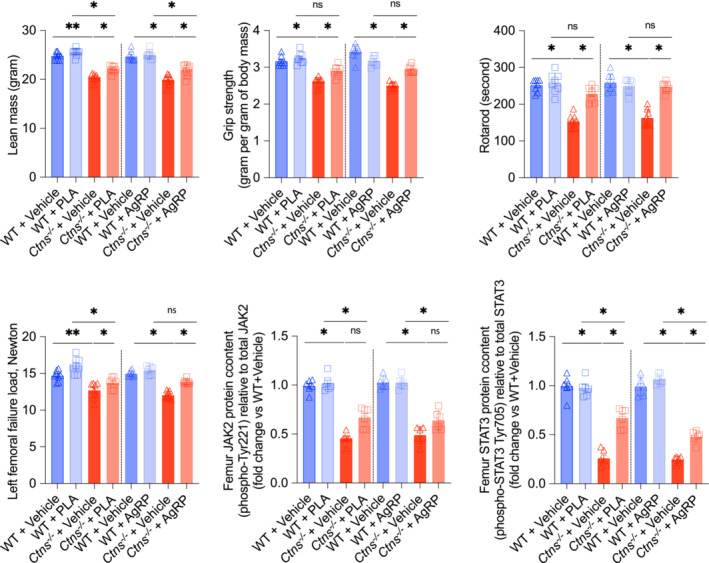
PLA and AgRP increase lean mass content, improve in vivo muscle function and femoral bone strength in *Ctns* ^*−/−*^ mice. Twelve‐month‐old *Ctns* ^*−/−*^ and WT mice were administered with PLA, AgRP, or vehicle, respectively. Figures 3 and 4 describe detailed study procedures. Lean mass content of individual mice was measured using quantitative magnetic resonance analysis. In vivo muscle function (grip strength and rotarod) was assessed. Femoral shaft biomechanical tests were performed. Femur protein contents of JAK2 and STAT3 were measured. Data are expressed as mean ± SEM. Results were analysed and expressed as in Figures 3 and 4. Ns, not significant, *P < 0.05, **P < 0.01.

The effect of melanocortin signalling blockade in *Ctns* ^*−/−*^ mice one bone phenotype was further tested via a pharmacological approach. AgRP is a naturally occurring antagonist for MC4R.^13^ We tested response to CNS AgRP injection in *Ctns* ^*−/−*^ mice. The study period is 28 days. We also applied a pair‐feeding strategy. Vehicle‐infused *Ctns* ^*−/−*^ mice were fed ad libitum whereas AgRP‐infused *Ctns* ^*−/−*^ mice, AgRP‐infused WT mice and vehicle‐infused WT mice were given the equivalent amount of energy intake as vehicle‐infused *Ctns* ^*−/−*^ mice (Figure 4). Results of serum and blood chemistry in *Ctns* ^*−/−*^ mice administered with AgRP are comparable to those in *Ctns* ^*−/−*^ mice infused with vehicle (Table 4). AgRP improved weight gain and whole‐body BMC/BMD in *Ctns* ^*−/−*^ mice (Figure 4). Femoral BMD was also significantly elevated in *Ctns* ^*−/−*^ mice treated with AgRP relative to *Ctns* ^*−/−*^ mice infused with vehicle. Effects of AgRP infusion on the femoral failure load in *Ctns* ^*−/−*^ mice are presented in the following Figure 5.

**Table 4:** Serum and blood chemistry of mice

	WT + Vehicle (*n* = 8)	WT + AgRP (*n* = 8)	*Ctns* ^*−/−*^ + Vehicle (*n* = 8)	*Ctns* ^*−/−*^ + AgRP (*n* = 8)
BUN (mg/dL)	31.6 ± 5.8	29.7 ± 3.4	54.1 ± 8.3^a^	63.7 ± 7.5^a^
Creatinine (mg/dL)	0.08 ± 0.01	0.09 ± 0.03	0.17 ± 0.03^a^	0.19 ± 0.03^a^
Bicarbonate (mg/dL)	27.5 ± 1.3	27.4 ± 0.5	27.7 ± 0.9	27.8 ± 0.5
Calcium (mg/dL)	9.45 ± 0.17	9.78 ± 0.28	9.49 ± 0.38	9.89 ± 0.45
Phosphorus (mg/dL)	9.23 ± 0.38	9.59 ± 0.39	8.02 ± 0.28^b^	7.48 ± 0.58^b^
Intact PTH (pg/mL)	89.3 ± 16.3	105.2 ± 18.8	385.8 ± 43.1^a^	351.7 ± 28.5^a^
FGF23 (pg/mL)	162.1 ± 24.3	132.6 ± 16.3	306.1 ± 24.3^a^	295.6 ± 34.2^a^

Twelve‐month‐old *Ctns* ^*−/−*^ mice and WT mice were infused with AgRP (2 nmol, intracranial) or vehicle (normal saline) at day 0, 3, 6, 9, 12, 15, 21, 24, and 27. All mice were sacrificed 1 day after the last dose of infusion. Four groups of mice were included: WT + vehicle, WT + AgRP, *Ctns* ^*−/−*^ + vehicle and *Ctns* ^*−/−*^ + AgRP. *Ctns* ^*−/−*^ + vehicle mice were fed ad libitum whereas WT + vehicle, WT + AgRP, and *Ctns* ^*−/−*^ + AgRP mice were pair‐fed to that of *Ctns* ^*−/−*^ + vehicle mice. Data are expressed as mean ± SEM. Data are expressed as mean ± SEM. Results of *Ctns* ^*−/−*^ + vehicle mice were compared with that of WT + vehicle mice whereas results of *Ctns* ^*−/−*^ + AgRP mice were compared with that of WT + AgRP mice.

^a^ *P* < 0.05, significantly higher in *Ctns* ^*−/−*^ mice than WT mice.

^b^ *P* < 0.05, significantly lower in *Ctns* ^*−/−*^ mice than WT mice.

### The pegylated leptin receptor antagonist and the agouti‐related peptide treatment improve muscle‐bone unit in *Ctns* ^−/−^ mice

Previous studies suggested that muscle mass is directly linked to bone health, building the concept of the ‘muscle‐bone unit’.^16^ We measured lean mass content and in vivo muscle function in mice. We found that PLA or AgRP significantly increased lean mass and improved muscle function (grip strength and rotarod activity) in *Ctns* ^*−/−*^ mice as compared with vehicle‐treated *Ctns* ^*−/−*^ mice (Figure 5). Additionally, we measured femoral whole‐bone strength (maximum failure load) by 3‐point bending. *Ctns* ^*−/−*^ mice treated with PLA or AgRP had significantly improved femoral failure load compared with *Ctns* ^*−/−*^ mice treated with vehicle. Recent data suggest that JAK/STAT signalling pathway is crucial for skeletal development and bone homeostasis. Decreased femur protein content of JAK2 and STAT3 was evident in vehicle‐treated *Ctns* ^*−/−*^ mice. PLA or AgRP attenuated STAT3 expression in femur of *Ctns* ^*−/−*^ mice.

## Discussion

The hormone leptin regulates bone metabolism. There is evidence that leptin influences bone metabolism via the hypothalamic melanocortin system.^6^, ^7^ In the arcuate nucleus of the hypothalamus, leptin inhibits neuropeptide Y (NPY) and agouti‐related peptide (AgRP) and enhances proopiomelanocortin (POMC) and cocaine‐ and amphetamine‐related transcript (CART).^6^ The leptin receptor, which can also be found in various peripheral organs and tissues, is responsible for leptin's actions.^6^ In fact, animal and human studies suggest that leptin influences bone metabolism in direct and indirect ways. In the human primary osteoblasts and chondrocytes, leptin receptors are found.^6^ FGF‐23, which is activated by leptin, may play a role in bone growth.^17^ Furthermore, leptin is also involved in the regulation of osteocalcin, which in turn is involved in the regulation of insulin, energy expenditure and bone metabolism.^18^ It is thought that bone metabolism may be modulated locally by leptin released from bone marrow adipocytes.^19^ Despite being an important regulator of bone metabolism, the effects of leptin on bone mass in rodents are contradictory. The bone phenotype of leptin‐deficient *ob/ob* mice is characterized by high bone mass. Following intracerebral leptin injection, lean mice and obese mice lose bone mass.^6^ *ob/ob* mice are also reported to have less bone mass than normal mice. The administration of intraperitoneal leptin to *ob/ob* mice increased cortical bone formation.^20^ Based on these conflicting findings, leptin appears to exert multiple effects depending on rodent skeletal maturity and signalling pathways.^6^, ^21^ We previously found that serum leptin levels are elevated in *Ctns* ^*−/−*^ mice, a model of INC.^9^ In this study, we sought to determine whether leptin signalling, which may involve the hypothalamic melanocortin pathway, contributed to pathogenesis of INC associated bone disease.

*Ctns* ^*−/−*^ mice showed lower whole‐body and femoral BMC/BMD than age‐matched WT mice (Figure 1). The results we report are consistent with those previously published.^22^ In patients with INC, phosphaturia leading to phosphate deficiency is thought to be the main cause of clinical manifestations of renal rickets in young INC patients.^2^ However, in early CKD stages, phosphaturia maintains serum phosphate concentrations thus protecting CKD patients from elevations of FGF‐23.^23^ In this study, serum phosphorus, FGF23, PTH, vitamin D metabolites, BUN, and creatinine concentrations were comparable between 1‐month‐old *Ctns* ^*−/−*^ mice and WT mice (Table 1). These data suggest that factors other than the known dysregulated bone mineral metabolism in CKD may contribute to the intrinsic bone defects in *Ctns* ^*−/−*^ mice. At 4 months, modest hypophosphataemia was the only significant difference among the bone mineral factors in *Ctns* ^*−/−*^ mice. At 9 and 12 months, *Ctns* ^*−/−*^ mice exhibited dysregulated bone mineral factors typically associated with classic CKD.

We investigated the impact of MC4R blockade on bone disease in *Ctns* ^*−/−*^ mice genetically. *Ctns* ^*−/−*^ *Mc4r* ^*−/−*^ mice, *Ctns* ^*−/−*^ mice, and WT controls were on the same C57BL/6 genetic background. We compared the bone phenotype of *Ctns* ^*−/−*^ *Mc4r* ^*−/−*^ mice, *Ctns* ^*−/−*^ mice, and WT controls. Femoral BMD, bone area, length and failure load of *Ctns* ^*−/−*^ *Mc4r* ^*−/−*^ mice were significantly increased compared with *Ctns* ^*−/−*^ mice at 9 months of age (Figure 2).

A pharmacological approach was then used to test whether leptin signalling accounts for bone defects in *Ctns* ^*−/−*^ mice. We tested two reagents, PLA, a pegylated leptin receptor antagonist, and AgRP, a competitive reverse agonist for MC4R. MC4R is expressed principally in the CNS, but bone cells also express this receptor.^24^ We treated 12‐month‐old *Ctns* ^*−/−*^ mice and WT controls with PLA or AgRP for 28 days respectively. We administered intracranial AgRP to isolate hypothalamic MC4R signalling. Our published results show that PLA or AgRP stimulate energy intake in mice.^9^, ^11^ Hence, a pair‐feeding strategy was applied to investigate the pharmacological effects of PLA or AgRP administration in *Ctns* ^*−/−*^ mice that goes beyond dietary stimulation and its accompanying weight gain. Vehicle‐injected *Ctns* ^*−/−*^ mice were fed ad libitum, and PLA‐injected, AgRP‐infused *Ctns* ^*−/−*^ mice or WT mice were pair‐fed with vehicle‐injected *Ctns* ^*−/−*^ mice. PLA significantly increased whole‐body and femoral BMC/BMD in *Ctns* ^*−/−*^ mice than in vehicle‐injected *Ctns* ^*−/−*^ mice (Figure 3). Similarly, whole‐body BMC/BMD/bone area and femoral BMD were significantly increased in AgRP‐infused *Ctns* ^*−/−*^ mice relative to vehicle‐infused *Ctns* ^*−/−*^ mice (Figure 4). Our results suggest that PLA or AgRP's protective effect on bone phenotype (whole‐body and femoral BMC/BMD) in *Ctns* ^*−/−*^ mice is independent of their known potent orexigenic effect. Moreover, these results suggest that leptin, possibly through a central hypothalamic mechanism, affects bone metabolism in *Ctns* ^*−/−*^ mice. We found no difference in femoral length in *Ctns* ^*−/−*^ mice, either treated with PLA or AgRP, compared with those *Ctns* ^*−/−*^ mice treated with vehicle (Figure 3I and Figure 4I). Postnatal development of the skeleton (especially long bones) continues until sexual maturity, at which point chondrocyte differentiation and apoptosis prevent growth of the growth plate.^25^ In previous studies, most skeletal maturity factors (including vertical length) in C57BL/6 mice occurred before 6 months of age.^26^ We used 12‐month‐old *Ctns* ^*−/−*^ mice (with a C57BL/6 genetic background) in this study as they already reached maximum vertical length at the time.

Lean mass content, muscle function and femoral whole‐bone strength were significantly improved in *Ctns* ^*−/−*^ mice treated with PLA or AgRP than vehicle‐treated *Ctns* ^*−/−*^ mice (Figure 5). Peripherally treatment of leptin antagonist significantly improved the mechanical properties of the tibia (maximal load, whole bone stiffness, and AUC) in female C57BL/6 mice.^27^ We examined femoral mechanical strength in mice as the femur is the strongest bone in the body because of its shape and size. With its thickness, the femur can withstand the pressure of the upper body and the pull of the muscles attached to it. In mice and humans, femoral BMC and BMD are significant predictors of peak loads in the femur.^28^ We measured lean mass content by EchoMRI and in vivo muscle function (grip strength and rotarod) in mice. Mice grip strength testing measures the maximal muscle strength of the forelimbs and hindlimbs together. Rotarod activity assesses motor function and coordination in rodents. Muscle and bone mass are strongly correlated. Muscles and bones are viewed as a functional unit, according to the mechanostat theory.^16^ Significant increases in grip strength for athletes (tennis and squash players) were associated with increased bone mass, BMD and bone area in the dominant arms compared with the non‐dominant arm.^29^ It is thought that mechanical forces derived from skeletal muscle stimulate bone formation and remodelling.^30^ There is evidence that muscles and bones communicate at a molecular level, in addition to mechanical coupling. Muscles and bones are interconnected, producing myokines (muscle‐derived factors) and osteokines (bone‐derived factors). Through autocrine, paracrine, and endocrine signalling, each factor influences metabolism of muscle and bone.^31^

Leptin signals are mediated via transmembrane receptors (Ob‐R) abundantly expressed in various tissues including bone. The activation of Ob‐R by leptin triggers multiple signalling pathways, including JAK/STAT signalling.^32^ The JAK/STAT pathway plays a crucial role in almost all tissues by orchestrating growth, differentiation and homeostasis. Recent data suggest that leptin signalling influences osteogenic differentiation of mesenchymal stem cells through JAK/STAT signalling.^33^ Zhou et al. have demonstrated the important role of STAT3 for bone development and bone homeostasis. They showed that pharmacologic activation of STAT3 attenuates bone deformities in mice while inhibition aggravates bone loss.^34^ We investigated the effect of PLA and AgRP treatment on JAK2 and STAT3 content in *Ctns* ^*−/−*^ mice femurs. A decrease in femur JAK2 and STAT3 protein levels was observed in *Ctns* ^*−/−*^ mice treated with vehicle (Figure 5). AgRP and PLA attenuated STAT3 expression in *Ctns* ^*−/−*^ mice femurs.

There are important implications for clinical practice associated with muscle‐bone crosstalk, including therapeutic targets that can improve muscle and bone health. As a result, INC patients may experience improved health and wellbeing overall. Strength of handgrip is one of the most significant metrics used to measure muscle strength and physical fitness. It is relevant to patients with sarcopenia, which is marked by decreased mass of muscles, diminished strength, and impaired function of the muscles.^35^ A multinational, large epidemiological study suggests that reduced muscular strength, as assessed by handgrip strength, indicates an elevated risk of death from all causes and cardiovascular disease in a prospective study in healthy adults.^36^ There is a significant reduction in grip strength in adults and children with INC, which was even more pronounced than in patients with CKD.^37^ Recently, we found that intraperitoneally injected PLA increased dietary intake, improved lean mass content, and improved muscle performance in *Ctns* ^*−/−*^ mice.^9^ By blocking leptin signalling in this study, PLA and AgRP improved the muscle‐bone unit in *Ctns* ^*−/−*^ mice (Figure 5). A limitation of the latter is the need for intraventricular delivery. A few potent peripheral MC4R antagonists have recently been discovered.^38^ A synthetic MC4R antagonist attenuates cachexia in cancer and CKD animal models.^39^ This newly developed MC4R antagonist has the potential to improve bone disease in *Ctns* ^*−/−*^ mice.

## Conclusions

We have employed both transgenic targeted mutant mouse models and pharmacological approaches to obtain further insight into the pathobiology of INC bone disease. We conclude that dysregulation of leptin signalling, either through the leptin receptor or through the melanocortin signalling system, is a significant contributor to INC‐related bone disease. Blocking leptin signalling may represent a novel therapeutic approach to this debilitating complication in INC.

## Conflict of interest

The authors declare no conflict of interest.

## Funding

This work was supported grants from the National Institutes of Health R01 DK125811 and the Cystinosis Research Foundation to R.H.M. This work was also supported by Young Investigator Grant of the National Kidney Foundation to W.W.C. P.Z. is supported by the following grants: 2022 Key R&D Plan of Science and Technology Department of Sichuan Province (project: 2022YFS0149); Science and Technology Fund of Chengdu Medical College in 2021 (project: CYZYB21‐22); Medical Research Project of Sichuan Province in 2021; Sichuan Medical Association (project: S21037); Medical Research Project of Chengdu Municipal Health Commission (project: 2022113); Health Commission of Sichuan Province Technology Projects in 2023; Clinical Research Projects (project: 23LCYJ015); and Cohort Study Project of the Institute's Science and Technology Innovation Fund of Sichuan Provincial Maternity and Child Health Care Hospital in 2023 (project: CXZD2023‐01).

## References

[1] Town MM , Jean G , Cherqui S , Attard M , Forestier L , Whitmore SA , et al. A novel gene encoding an integral membrane protein is mutated in nephropathic cystinosis. Nat Genet 1998;18:319–324. 9537412 10.1038/ng0498-319

[2] Gahl W , Thoene JG , Schneider JA . Cystinosis. N Engl J Med 2002;347:111–121. 12110740 10.1056/NEJMra020552

[3] Machuca‐Gayet I , Quinauz T , Bertholet‐Thomas A , Gaillard S , Claramunt‐Taberner D , Acquaviva‐Bourdain C , et al. Bone disease in nephropathic cystinosis: beyond renal osteodystrophy. Int J Mol Sci 2020;21:3109. 32354056 10.3390/ijms21093109 PMC7246679

[4] Florenzano P , Ferreira C , Nesterova G , Roberts MS , Tella SH , de Castro LF , et al. Skeletal consequences of nephropathic cystinosis. J Bone Miner Res 2018;33:1870–1880. 29905968 10.1002/jbmr.3522 PMC13186320

[5] Hohenfellner K , Rauch F , Ariceta G , Awan A , Bacchetta J , Bergmann C , et al. Management of bone disease in cystinosis: statement from an international conference. J Inherit Metab Dis 2019;42:1019–1029. 31177550 10.1002/jimd.12134 PMC7379238

[6] Upadhyay J , Farr OM , Mantzoros CS . The role of leptin in regulating bone metabolism. Metabolism 2015;64:105–113. 25497343 10.1016/j.metabol.2014.10.021 PMC4532332

[7] Ducy P , Amling M , Takeda S , Priemel M , Schilling AF , Beil FT , et al. Leptin inhibits bone formation through a hypothalamic relay: a central control of bone mass. Cell 2000;100:197–207. 10660043 10.1016/s0092-8674(00)81558-5

[8] Mak RH , Cheung W , Cone RD , Marks DL . Leptin and inflammation‐associated cachexia in chronic kidney disease. Kidney Int 2006;69:794–797. 16518340 10.1038/sj.ki.5000182

[9] Gonzalez A , Cheung W , Perens EA , Oliveira EA , Gertler A , Mak RH . A leptin receptor antagonist attenuates adipose tissue browning and muscle wasting in infantile nephropathic cystinosis‐associated cachexia. Cells 2021;10:1954. 34440723 10.3390/cells10081954 PMC8393983

[10] Farooqi IS , Yeo GSH , Keogh JM , Aminian S , Jebb SA , Butler G , et al. Dominant and recessive inheritance of morbid obesity associated with melanocortin 4 receptor deficiency. J Clin Invest 2000;106:271–279. 10903343 10.1172/JCI9397 PMC314308

[11] Cheung W , Yu PX , Little BM , Cone RD , Marks DL , Mak RH . Role of leptin and melanocortin signaling in uremia‐associated cachexia. J Clin Invest 2005;115:1659–1665. 15931394 10.1172/JCI22521 PMC1136984

[12] Elinav E , Niv‐Spector L , Katz M , Price TO , Ali M , Yacobovitz M , et al. Pegylated leptin antagonist is a potent orexigenic agent: preparation and mechanism of activity. Endocrinology 2009;150:3083–3091. 19342450 10.1210/en.2008-1706 PMC2703547

[13] Dhillo WS , Bloom SR . Hypothalamic peptides as drug targets for obesity. Curr Opin Pharmacol 2011;1:651–655. 10.1016/s1471-4892(01)00110-2 11757822

[14] Cheung WW , Cherqui S , Ding W , Esparza M , Zhou P , Shao J , et al. Muscle wasting and adipose tissue browning in infantile nephropathic cystinosis. J Cachexia Sarcopenia Muscle 2015;7:152–164. 27493869 10.1002/jcsm.12056 PMC4864942

[15] Janssens V , Chevronnay HPG , Marie S , Vincent MF , Van Der Smissen P , Nevo N , et al. Protection of cystinotic mice by kidney‐specific megalin ablation supports an endocytosis‐based mechanism for nephropathic cystinosis progression. J Am Soc Nephrol 2019;30:2177–2190. 31548351 10.1681/ASN.2019040371 PMC6830792

[16] Haffner D , Leifheit‐Nestler M , Alioli C , Bacchetta J . Muscle and bone impairment in infantile nephropathic cystinosis: new concepts. Cells 2022;11:170. 35011732 10.3390/cells11010170 PMC8749987

[17] Tsuji K , Maeda T , Kawane T , Matsunuma A , Horiuchi N . Leptin stimulates fibroblast growth factor 23 expression in bone and suppresses renal 1 alpha,25‐dihydroxyvitamin d3 synthesis in leptin‐deficient mice. J Bone Miner Res 2010;25:1711–1723. 20200981 10.1002/jbmr.65

[18] Ferron M , Lacombe J . Regulation of energy metabolism by the skeleton: osteocalcin and beyond. Arch Biochem Biophys 2014;561:137–146. 24893146 10.1016/j.abb.2014.05.022

[19] Laharrague R , Larrouy D , Fontanilles AM , Truel N , Campfield A , Tenenbaum R , et al. High expression of leptin by human bone marrow adipocytes in primary culture. FASEB J 1998;12:747–752. 9619453 10.1096/fasebj.12.9.747

[20] Hamrick MW , Della‐Fera MA , Choi YH , Pennington C , Hartzell D , Baile CA . Leptin treatment induces loss of bone marrow adipocytes and increases bone formation in leptin‐deficient ob/ob mice. J Bone Miner Res 2005;20:994–1001. 15883640 10.1359/JBMR.050103

[21] Maor G , Rochwerger M , Segev Y , Phillip M . Leptin acts as a growth factor on the chondrocytes of skeletal growth centers. J Bone Miner Res 2002;17:1034–1043. 12054158 10.1359/jbmr.2002.17.6.1034

[22] Battafarano G , Rossi M , Rega L , Di Giovamberardino G , Pastore A , D'Agostini M , et al. Intrinsic bone defects in cystinotic mice. Am J Pathol 2019;189:1053–1064. 30794806 10.1016/j.ajpath.2019.01.015

[23] Florenzano P , Jimenez M , Ferreira CR , Nesterova G , Roberts MS , Tella SH , et al. Nephropathic cystinosis: a distinct form of CKD‐mineral and bone disorder that provides novel insights into the regulation of FGF23. J Am Soc Nephrol 2020;31:2184–2192. 32631973 10.1681/ASN.2019111172 PMC7461669

[24] Dumont LM , Wu CSJ , Tatnell MA , Cornish J , Mountjoy KG . Evidence for direct actions of melanocortin peptides on bone metabolism. Peptides 2005;26:1929–1935. 15979763 10.1016/j.peptides.2004.12.034

[25] Hallett SA , Ono W , Ono N . Growth plate chondrocytes: skeletal development, growth and beyond. Int J Mol Sci 2019;29:6009. 10.3390/ijms20236009 PMC6929081 31795305

[26] Somerville JM , Aspden RM , Armour KE , Armour KJ , Reid DM . Growth of C57Bl/6 mice and the material and mechanical properties of cortical bone from the tibia. Calcif Tissue Int 2004;74:469–475. 14961209 10.1007/s00223-003-0101-x

[27] Solomon G , Atkins A , Shahar R , Gertler A , Monsonego‐Ornan E . Effect of peripherally administered leptin antagonist on whole body metabolism and bone microarchitecture and biomechanical properties in the mouse. Am J Physiol Endocrinol Metab 2014;306:E14–E27. 24169045 10.1152/ajpendo.00155.2013

[28] Han H , Chen S , Wang X , Jin J , Li X , Li Z . Association between muscle strength and mass and bone mineral density in the US general population: data from NHANES 1999–2002. J Orthop Surg Res 2023;18:397. 37264353 10.1186/s13018-023-03877-4 PMC10233893

[29] Kannus P , Haapasalo H , Sankelo M , Sievänen H , Pasanen M , Heinonen A , et al. Effect of starting age of physical activity on bone mass in the dominant arm of tennis and squash players. Ann Intern Med 1995;123:27–31. 7762910 10.7326/0003-4819-123-1-199507010-00003

[30] Schoenau E , Frost HM . The ‘muscle‐bone unit’ in children and adolescents. Calcif Tissue Int 2002;70:405–407. 11960207 10.1007/s00223-001-0048-8

[31] Wong L , McMahon LP . Crosstalk between bone and muscle in chronic kidney disease. Front Endocrinol (Lausanne) 2023;14:1146868. 37033253 10.3389/fendo.2023.1146868 PMC10076741

[32] Damerau A , Gaber T , Ohrndorf S , Hoff P . JAK/STAT activation: a general mechanism for bone development, homeostasis, and regeneration. Int J Mol Sci 2021;21:9004. 10.3390/ijms21239004 PMC7729940 33256266

[33] Zhang B , Yang L , Zeng Z , Feng Y , Wang X , Wu X , et al. Leptin potentiates BMP9‐induced osteogenic differentiation of mesenchymal stem cells through the activation of JAK/STAT signaling. Stem Cells Dev 2020;29:0292–0510. 10.1089/scd.2019.0292 PMC7153647 32041483

[34] Zhou S , Dai Q , Huang X , Jin A , Yang Y , Gong X , et al. STAT3 is critical for skeletal development and bone homeostasis by regulating osteogenesis. Nat Commun 2021;12:6891. 34824272 10.1038/s41467-021-27273-w PMC8616950

[35] Vaishya R , Misra A , Vaish A , Ursino N , D'Ambrosi R . Hand grip strength as a proposed new vital sign of health: a narrative review of evidences. J Health Popul Nutr 2024;43:7. 38195493 10.1186/s41043-024-00500-y PMC10777545

[36] Leong DP , Teo KK , Rangarajan S , Lopez‐Jaramillo P , Avezum A Jr , Orlandini A , et al. Prognostic value if grip strength: findings form the Prospective Urban Rural Epidemiology (PURE) study. Lancet 2015;386:266–273. 25982160 10.1016/S0140-6736(14)62000-6

[37] Lyob‐Tessema H , Wang CS , Kennedy S , Reyes L , Shin S , Greenbaum L , et al. Grip strength in adults and children with cystinosis. Kidney Int Rep 2021;6:389–395. 33615064 10.1016/j.ekir.2020.11.017 PMC7879123

[38] Garnsey MR , Smith AC , Polivkova J , Arons AL , Bai G , Blakemore C , et al. Discovery of the potent and selective MC4R antagonist PF‐07258669 for the potential treatment of appetite loss. J Med Chem 2023;66:3195–3211. 36802610 10.1021/acs.jmedchem.2c02012

[39] Zhu X , Callahan MF , Gruber KA , Szumowski M , Marks DL . Melanocortin‐4 receptor antagonist TCMCB07 ameliorates cancer‐ and chronic kidney disease–associated cachexia. J Clin Invest 2020;130:4921–4934. 32544087 10.1172/JCI138392 PMC7456235

